# Syndromes with short stature

**DOI:** 10.1186/1824-7288-41-S2-A73

**Published:** 2015-09-30

**Authors:** Luigi Tarani, Francesca Mancini, Natascia Liberati, Giovanni Parlapiano, Leonardo Pimpolari, Michela Martini, Chiara Mancini, Fiorenza Colloridi

**Affiliations:** 1 Department of Pediatrics and Pediatric Neuropsychiatry, “Sapienza”, University of Rome, Rome, Italy

## 

At present, factors that have been recognized as being able to influence growth are: nutritional, physical, chemical, psychological and genetic.

The causes of short stature are numerous, with about 90% of cases classified as Idiopathic Short Stature and divided into 2 types familial short stature and constitutional short stature.

Part of the population with growth disorders are SGA newborns (10%) who don't show recovery growth that physiologically should occur within 2-3 years in 90% of cases.

Generally in this group there are both children small due to severe prematurity and children afflicted by genetic syndromes.

Syndromes are the subject of our interest and we thought it would be useful to distinguish different prenatal and postnatal patterns of growth. In fact, some specific patterns can characterize syndromes. For example, very slow prenatal and postnatal growth is typical of Silver-Russell Syndrome; low prenatal and childhood growth followed by obesity after 3 years of life is typical of Prader–Willi syndrome; prenatal and postnatal overgrowth is typical of Beckwith–Wiedemann syndrome; and prenatal overgrowth followed by low postnatal growth is typical of Costello syndrome.

Short stature can be harmonious or disharmonious; harmonious ones include familiar short stature, constitutional growth delays, GH deficiency, intrauterine growth restriction, dysmorphic or genetic syndrome and short stature in the presence of chronic diseases.

In this paper we discuss the growth patterns of some genetic syndromes, such as achondroplasia, CHARGE syndrome, Cornelia de Lange syndrome, 22q11.2 deletion syndrome, Downs syndrome, Fetal – alcohol syndrome, Kabuki syndrome, Noonan syndrome, Prader-Willi syndrome, Rubistein–Taybi syndrome, Silver–Russell syndrome, Turner syndrome, Williams syndrome and VACTERL/VATER association.

